# Factors associated with clinical research participation among Medicare beneficiaries with prostate cancer in the United States

**DOI:** 10.3389/fonc.2026.1887816

**Published:** 2026-09-03

**Authors:** Ryan A. Hankins, Alysha M. McGovern, Abimbola O. Williams, Virgil H. Simons, Sean P. Collins, Deepak Kumar

**Affiliations:** 1 Department of Urology, MedStar Georgetown University Hospital, Washington, DC, United States; 2 Health Economics and Market Access, Boston Scientific, Marlborough, MA, United States; 3 The Prostate Net, Inc., Sanford, NC, United States; 4 University of South Florida (USF) Health Morsani College of Medicine, Tampa, FL, United States; 5 Julius L. Chambers Biomedical/Biotechnology Research Institute, Department of Pharmaceutical Sciences, North Carolina Central University, Durham, NC, United States

**Keywords:** clinical trial diversity, disparities in care, health equity, prostate cancer, social determinants of health

## Abstract

**Introduction:**

Clinical research participation among patients with prostate cancer remains limited, and demographic, clinical, and contextual factors may influence opportunities for participation. Although disparities in prostate cancer diagnosis and treatment are well documented, less is known about the factors associated with clinical research participation in this population. This study examined patient-level and contextual factors associated with clinical research participation among older Medicare beneficiaries with prostate cancer.

**Methods:**

A retrospective claims-based analysis was conducted using the Medicare 5% Standard Analytical Files to identify men aged ≥65 years with a healthcare encounter for prostate cancer between January 1, 2016 and December 31, 2021. Clinical research participation was identified using the International Classification of Diseases, Tenth Revision, Clinical Modification diagnosis code Z00.6 (“Encounter for examination for normal comparison and control in clinical research program”). Deidentified patient-level data were linked to 2020 county-level social determinants of health (SDOH) data from the Agency for Healthcare Research and Quality database. Multivariable logistic regression models were used to evaluate demographic, clinical, and county-level contextual factors associated with clinical research participation.

**Results:**

A total of 107,250 Medicare Fee-for-Service (FFS) beneficiaries with prostate cancer were identified, representing approximately 2.145 million FFS beneficiaries after standard extrapolation. Among this cohort, 4,361 (4.07%) beneficiaries had documented clinical research participation, corresponding to approximately 87,220 beneficiaries after extrapolation. Compared to beneficiaries without documented research participation, beneficiaries participating in clinical research were younger, more often White, had higher Charlson Comorbidity Index (CCI) scores, and higher rates of metastatic disease. Residence in counties with higher socioeconomic indicators, including higher median household income, lower unemployment, higher educational attainment, and greater internet access was also associated with clinical research participation. After controlling for age, race, and CCI score, adjusted analyses identified several patient-level clinical and geographic characteristics, as well as county-level contextual characteristics, associated with clinical research participation, including metastatic disease, census region, county-level median household income, and county-level median distance to the nearest emergency department.

**Discussion:**

These findings suggest that patient- and community-level factors may influence participation in clinical research. Targeted efforts to reduce structural and geographic barriers may help promote more equitable participation in clinical research among prostate cancer patients.

## 1 Introduction

Prostate cancer is one of the most commonly diagnosed malignancies among men in the United States (U.S.), with approximately 1 in 8 men diagnosed with prostate cancer during their lifetime (1, 2). It remains a leading cause of cancer-related mortality (1), posing a substantial burden on both patients and the U.S. healthcare system. Clinical research plays a critical role in advancing cancer care by facilitating the development and approval of novel diagnostic, therapeutic, and supportive care interventions that improve patient care and outcomes (3). However, participation in oncology clinical trials remains limited and demographically skewed, potentially limiting the generalizability of research findings and equitable access to emerging therapies (4–10).

Overall, fewer than 8% of adults with cancer in the U.S. participate in clinical trials (11). Participation is particularly limited among older adults, who represent a majority of cancer diagnoses but have historically been underrepresented in cancer research, enrolling in clinical trials at rates as low as 1.0% to 1.9% (12). Among men with prostate cancer, trial participation has been reported as low as 1% or less (8), raising concerns about the representativeness of evidence used to guide care in this population. Previous studies have also documented that barriers to participation are multifactorial and include factors such as individual-level demographic and clinical characteristics, as well as structural and contextual social determinants of health (SDOH), such as socioeconomic status, access to care, and geographic location (11).

Low and unequal participation in clinical research has important implications for cancer care. Underrepresentation of certain populations may limit the generalizability of research findings and hinder the equitable delivery of innovative cancer therapies. Participation in trials has been associated with improved short-term survival in cancer populations (13), further emphasizing the importance of equitable enrollment in clinical trials. Additionally, well-documented disparities in prostate cancer diagnosis, treatment, and outcomes by race and socioeconomic status (14, 15) underscore the importance of ensuring research populations reflect the broader spectrum of affected individuals.

Despite increasing attention to improving diversity in clinical trials (16–18) and growing recognition of multilevel barriers to participation (19–22), limited evidence exists regarding the individual- and community-level factors associated with claims-identified clinical research participation among older adults with prostate cancer. Therefore, this study aimed to address this gap by examining both patient- and county-level factors associated with claims-identified clinical research participation among U.S. Medicare Fee-for-Service (FFS) beneficiaries with a prostate cancer diagnosis.

## 2 Materials and methods

### 2.1 Study type and patient population

A retrospective cohort study was conducted using the Medicare 5% Standard Analytical Files (SAF), which include information on beneficiary enrollment, claims from healthcare encounters, such as diagnoses and procedures, and demographic information, such as age, sex, and race, from a 5% random sample of all Medicare FFS beneficiaries.

The study population consisted of male Medicare FFS beneficiaries aged 65 years and older with at least one healthcare encounter for prostate cancer between January 1, 2016 and December 31, 2021. Prostate cancer was identified using International Classification of Diseases, Tenth Revision, Clinical Modification (ICD-10-CM) diagnosis code C61 (“Malignant neoplasm of prostate”). The date of the first recorded prostate cancer encounter was defined as the index date. To ensure sufficient baseline and follow-up data, eligible beneficiaries were required to have at least 12 months of continuous Medicare FFS enrollment both before and after the index date, unless follow-up was censored by death.

Beneficiaries were stratified by documented clinical research participation, defined by the presence of at least one claim containing ICD-10-CM diagnosis code Z00.6 (“Encounter for examination for normal comparison and control in clinical research program”) at any time following the index prostate cancer diagnosis. ICD-10-CM code Z00.6 is used to identify healthcare encounters associated with clinical research programs and is required for services provided as part of a clinical research program, regardless of whether those services would otherwise be delivered as standard care for that patient (23). Although the code indicates that a research-related encounter occurred, it does not distinguish prostate cancer-specific studies from clinical research involving other conditions and does not identify the type, phase, or duration of the research program. Accordingly, the outcome was interpreted as claims-identified clinical research participation among Medicare FFS beneficiaries with prostate cancer rather than confirmed participation in prostate cancer-specific trials.

For descriptive purposes, patient counts from the Medicare 5% sample were multiplied by 20 using the standard extrapolation factor to approximate the Medicare FFS population. These extrapolated counts should be interpreted as approximate estimates of the Medicare FFS population and not as calibrated national estimates.

To evaluate SDOH factors, beneficiary records were linked to 2020 county-level data from the Agency for Healthcare Research and Quality (AHRQ) SDOH Database (24). This database integrates information from more than 40 publicly available data sources spanning 2009 to 2020, including the American Community Survey, Area Health Resource Files, County Health Rankings, and the National Center for Health Statistics. It contains comprehensive data across five key SDOH domains: social context (demographics, living conditions, disability, immigration, socioeconomic disadvantage indices, segregation), economic context (income, employment, poverty), education (attainment, school system, educational funding, literacy, numeracy), physical infrastructure (housing, transportation, migration, internet connectivity, environment, industry composition, social services, food access, access to exercise, crime), and healthcare context (health insurance status, characteristics of healthcare providers and facilities, distance to provider, utilization and costs, health behaviors and outcomes, healthcare equity). County-level SDOH measures were assigned according to beneficiaries’ county of residence and were evaluated as contextual characteristics rather than individual-level measures.

### 2.2 Outcomes and measures

The primary outcome was documented clinical research participation, categorized as a binary variable (yes/no). Demographic, clinical, and county-level SDOH characteristics were compared between beneficiaries with and without documented clinical research participation to identify factors associated with clinical research participation.

Patient-level demographic and clinical variables derived from the Medicare SAF included age at prostate cancer diagnosis, race, census region, presence of metastatic disease (defined using ICD-10 diagnosis codes; see **Supplementary Table 1**), and comorbidity burden measured using the Charlson Comorbidity Index (CCI) (25). County-level measures from all five domains of the AHRQ SDOH database were evaluated as contextual characteristics. Social domain variables included urbanicity, citizenship and birthplace, racial composition, and Area Deprivation Index (ADI). Economic and education domain variables included median household income, per capita income, unemployment, educational attainment, and poverty. Physical infrastructure variables included median rent, median home values, the percentage of residents receiving public assistance, and the percentage of households without access to a vehicle or internet. Healthcare domain variables included the percentage of uninsured individuals, Medicaid enrollment rates, and median distance in miles to the nearest emergency department (ED) or urgent care facility, calculated using population-weighted tract centroids in the county. County-level SDOH measures were assigned to beneficiaries based on their county of residence and interpreted as contextual characteristics rather than individual-level measures.

### 2.3 Statistical analyses

Descriptive statistics were used to summarize the study population overall and by clinical research participation. Categorical variables were summarized as frequencies and proportions, while continuous variables were summarized using means, standard deviations (SDs), and medians. Comparisons between beneficiaries with and without documented clinical research participation were conducted using chi-square tests for categorical variables and t-tests for continuous variables. Statistical significance was defined as a p-value of less than 0.05.

Multivariable logistic regression analyses were conducted to evaluate patient- and county-level factors associated with clinical research participation. Both unadjusted and adjusted models were estimated. Patient-level covariates of age, race, and CCI score were selected *a priori* as the base adjustment variables based on clinical relevance and published literature evaluating factors associated with clinical research participation. Separate adjusted models were then estimated to evaluate the associations between clinical research participation and individual clinical or county-level contextual characteristics, including census region, metastatic disease, and select county-level SDOH measures. County-level SDOH measures were selected to represent multiple contextual domains, including socioeconomic conditions, educational attainment, physical infrastructure, and healthcare accessibility. When multiple variables reflected similar constructs within a domain, representative measures were selected based on their established use in prior literature and their ability to represent the broader contextual domain. Because many county-level SDOH measures capture related aspects of community context and are conceptually interrelated, each SDOH measure was evaluated individually while adjusting for the same prespecified patient-level base covariates. This approach was intended to characterize associations between individual contextual domains and documented clinical research participation rather than estimate the independent effects of multiple correlated county-level variables entered simultaneously. Accordingly, associations observed across separate models should not be interpreted as independent effects of multiple contextual domains. Given the number of associations evaluated, individual statistically significant findings should be interpreted in the context of the overall pattern of results and their consistency with prior literature. A final adjusted model was then estimated including age, race, CCI score, census region, metastatic disease, and selected-county level variables representing distinct contextual domains to evaluate their simultaneous associations with clinical research participation. To facilitate the interpretability of adjusted odds ratios (aORs), continuous county-level household income and median home value variables were rescaled in $10,000 increments prior to regression modeling.

All analyses were performed using the Instant Health Data software (Panalgo, Boston, MA, USA) and Stata version 18.0 (StataCorp, College Station, TX, USA).

## 3 Results

A total of 107,250 Medicare FFS beneficiaries with prostate cancer were identified in the database, corresponding to approximately 2,145,000 Medicare FFS beneficiaries after standard extrapolation. Among these, 4,361 (4.07%) beneficiaries had at least one claim containing ICD-10-CM diagnosis code Z00.6 (**Table 1**), corresponding to approximately 87,220 beneficiaries after extrapolation.

**Table 1:** Demographic, clinical, and county-level social determinants of health characteristics by documented clinical research participation.

Characteristics	No documented clinical research participation (n=102,889)	Documented clinical research participation (n=4,361)	P-value	Notes
Social context				
Age (years), mean (SD)	75.3 (7.3)	74.8 (6.4)	<0.01	
CCI Score, mean (SD)	2.05 (2.26)	2.52 (2.44)	<0.01	
Metastatic disease, n (%)	18,605 (18.1%)	1,395 (32.0%)	<0.01	
Race, n (%)			<0.01	
White	84,405 (82.0%)	3,891 (89.2%)		
Black	11,624 (11.3%)	261 (6.0%)		
Other/Unknown	6,860 (6.7%)	209 (4.8%)		
Census Region, n (%)			<0.01	
Midwest	22,198 (21.7%)	902 (20.7%)		
Northeast	20,576 (20.1%)	837 (19.2%)		
South	39,800 (38.8%)	1,646 (37.8%)		
West	19,921 (19.4%)	974 (22.3%)		
Urbanicity, n (%)			<0.01	Based on county Rural-Urban Continuum Code (RUCC)
Metropolitan	83,982 (81.6%)	3,714 (85.2%)		
Non-Metropolitan	18,907 (18.4%)	647 (14.8%)		
Foreign Born, mean (SD)	12.9% (10.4%)	13.8% (10.3%)	<0.01	Percentage of population in county who are foreign-born
Non-Citizen, mean (SD)	5.8% (4.7%)	6.2% (4.7%)	<0.01	Percentage of population in county who are not US citizens
White Population, mean (SD)	72.7% (16.7%)	73.0% (15.6%)	0.42	Percentage of population in county reporting White race alone
ADI, mean (SD)	52.7 (20.6)	50.7 (20.0)	<0.01	Based on 2020 national ADI ranking (higher ranking indicates more disadvantaged)
Economic context				
Median Household Income, mean (SD)	$67,292 ($19,043)	$69,613 ($18,791)	<0.01	County-level median household income, inflation-adjusted
Median Household Income Quintile, n (%)			<0.01	
Q1: ≤ $27,030	402 (0.4%)	*		
Q2: $27,031-$52,180	21,059 (20.5%)	670 (15.4%)		
Q3: $52,181-$85,080	64,189 (62.4%)	2,859 (65.6%)		
Q4: $85,081-$114,110	14,557 (14.2%)	702 (16.1%)		
Q5: $114,111+	2,682 (2.6%)	*		
Per Capita Income, mean (SD)	$35,451 ($9,431)	$36,599 ($9,369)	<0.01	County-level per capita income, inflation-adjusted
Unemployed, mean (SD)	5.4% (1.7%)	5.3% (1.5%)	<0.01	Percentage of population in county who are unemployed (ages 16+)
Poverty, mean (SD)	11.8% (4.3%)	11.3% (4.0%)	<0.01	Estimated percentage of people of all ages in poverty in county
Education				
Less than HS Education, mean (SD)	11.0% (4.7%)	10.8% (4.5%)	<0.01	Percentage of population in county with less than high school education (ages 25+)
Post-HS Education, mean (SD)	61.5% (9.5%)	62.6% (9.1%)	<0.01	Percentage of population in county with any postsecondary education (ages 25+)
Graduate Degree, mean (SD)	12.5% (5.7%)	13.0% (5.7%)	<0.01	Percentage of population in county with master’s or professional school degree or doctorate (ages 25+)
Limited English, mean (SD)	4.0% (5.5%)	4.0% (4.0%)	0.50	Percentage of limited English speaking households in county
No English, mean (SD)	1.2% (2.6%)	1.1% (1.5%)	0.16	Percentage of population that speaks a language other than English that does not speak English at all (ages 5+)
Physical infrastructure				
Median Rent, mean (SD)	$1,102 ($349)	$1,146 ($354)	<0.01	Median gross rent in county
Median Home Value, mean (SD)	$273,136 ($176,894)	$291,098 ($184,054)	<0.01	Median home value of owner-occupied housing units in county
Median home value grouping, n (%)				
Under $355,900	81,696 (79.4%)	3,319 (76.1%)	<0.01	
$355,900+	21,193 (20.6%)	1,042 (23.9%)		
Housing 10+ Units, mean (SD)	12.7% (10.9%)	13.2% (10.6%)	<0.01	Percentage of county housing in structures with 10 or more units
No Vehicle, mean (SD)	8.1% (8.4%)	7.6% (7.9%)	<0.01	Percentage of households in county with no vehicle
No Internet, mean (SD)	12.2% (5.6%)	11.4% (5.1%)	<0.01	Percentage of households in county with no internet access
Food Stamps, mean (SD)	11.2% (5.3%)	10.6% (4.8%)	<0.01	Percentage of households in county that received food stamps/SNAP, past 12 months
Public Assistance, mean (SD)	12.0% (5.4%)	11.3% (4.8%)	<0.01	Percentage of households in county with public assistance income or food stamps/SNAP
Assault Death, mean (SD)	6.6 (5.7)	6.1 (5.3)	<0.01	Total number of deaths from assault per 100,000 population in county
Opioid Deaths, mean (SD)	18.0 (11.9)	17.6 (11.4)	0.02	Total number of drug overdose deaths involving any opioid per 100,000 population in county
Healthcare context				
Uninsured, mean (SD)	8.4% (4.0%)	8.3% (4.0%)	0.34	Percentage of population in county with no health insurance coverage
Medicaid, mean (SD)	16.7% (6.6%)	16.1% (6.2%)	<0.01	Percentage of population in county with any Medicaid/means-tested public health insurance coverage
Median Distance to ED (miles) Category, n (%)				Median distance in miles to the nearest ED, calculated using population-weighted tract centroids in the county
Less than 4.0 miles	70,283 (68.3%)	3,065 (70.3%)	<0.01	
4.0 miles or greater	32,606 (31.7%)	1,296 (29.7%)		
Median Distance to Urgent Care (miles), mean (SD)	7.38 (12.0)	6.40 (11.1)	<0.01	Median distance in miles to the nearest urgent care, calculated using population-weighted tract centroids in the county

CCI, Charlson Comorbidity Index; ADI, Area Deprivation Index; HS, High School; ED, Emergency Department; SD, Standard Deviation.

* Per Medicare requirements, results with <11 outcomes were suppressed to ensure confidentiality.

Beneficiaries with documented clinical research participation were slightly younger (mean age 74.8 vs. 75.3 years, p<0.01) and differed significantly by race distribution (p<0.01), with a higher proportion of beneficiaries identified as White (89.2% vs. 82.0%, p<0.01) compared to those without documented clinical research participation. Clinical research participants also had a higher mean CCI score (2.52 vs. 2.05, p<0.01) and were significantly more likely to present with metastatic disease (32.0% vs. 18.1%, p<0.01, **Table 1**).

Geographically, clinical research participants were more likely to reside in metropolitan areas (85.2% vs. 81.6%, p<0.01) and in counties with increased socioeconomic indicators. Compared to beneficiaries without documented clinical research participation, clinical research participants resided in counties with higher median household income ($69,613 vs. $67,292, p<0.01), higher per capita income ($36,599 vs. $35,451, p<0.01), lower unemployment rates (5.3% vs. 5.4%, p<0.01), and lower poverty levels (11.3% vs. 11.8%, p<0.01, **Table 1**).

Additionally, clinical research participants more frequently resided in counties characterized by higher levels of educational attainment, greater median home values and rental costs, lower rates of violent assaults and opioid-related deaths, and better physical infrastructure, reflected in greater vehicle and internet access (**Table 1**). County-level healthcare access measures also differed, with clinical research participants residing in counties with shorter median distances to emergency departments and urgent care facilities (**Table 1**). Conversely, beneficiaries without documented clinical research participation more frequently resided in counties with higher ADI scores, indicating greater socioeconomic and neighborhood disadvantage (**Table 1**).

In adjusted models, younger age, White race, and higher CCI score were consistently associated with greater odds of documented clinical research participation (**Table 2**). Models incorporating additional patient- and county-level measures identified additional factors associated with clinical research participation, including metastatic disease (aOR = 2.000, p<0.001), census region (aOR for West compared to Midwest = 1.220, p<0.001), metropolitan residence (aOR for non-metropolitan areas = 0.742, p<0.001), higher county-level median household income (aOR 1.067 per $10,000 increase, p<0.001), higher county-level median home value (aOR = 1.007 per $10,000 increase, p<0.001), greater county-level educational attainment (aOR = 1.014 for populations with education beyond high school, p<0.001), and shorter county-level distance to the nearest ED (aOR = 0.973 per mile, p<0.001, **Table 2**). In the adjusted model including multiple selected patient- and county-level covariates, age, race, CCI score, census region, metastases, median household income, and distance to the nearest ED remained significantly associated with clinical research participation (**Table 2**).

**Table 2:** Adjusted associations between patient- and county-level characteristics and documented clinical research participation.

	Region/metastatic disease		Metropolitan status		Household income		Educational attainment		Distance to ED		Home value		Final adjusted model	
Covariate	aOR (95% CI)	P-value	aOR (95% CI)	P-value	aOR (95% CI)	P-value	aOR (95% CI)	P-value	aOR (95% CI)	P-value	aOR (95% CI)	P-value	aOR (95% CI)	P-value
Age (continuous)	0.982 (0.977-0.986)	<0.001	0.982 (0.978-0.986)	<0.001	0.982 (0.978-0.986)	<0.001	0.982 (0.978-0.986)	<0.001	0.982 (0.978-0.986)	<0.001	0.982 (0.977-0.986)	<0.001	0.981 (0.977-0.986)	<0.001
White race (versus non-White)	1.886 (1.710-2.080)	<0.001	1.927 (1.748-2.124)	<0.001	1.889 (1.713-2.082)	<0.001	1.886 (1.711-2.079)	<0.001	1.930 (1.750-2.129)	<0.001	1.941 (1.760-2.140)	<0.001	1.932 (1.751-2.132)	<0.001
CCI score (continuous)	1.061 (1.048-1.074)	<0.001	1.095 (1.082-1.108)	<0.001	1.096 (1.083-1.110)	<0.001	1.098 (1.084-1.111)	<0.001	1.095 (1.082-1.108)	<0.001	1.096 (1.083-1.109)	<0.001	1.062 (1.049-1.075)	<0.001
Census region (versus Midwest)														
Northeast	1.026 (0.932-1.130)	0.597											0.931 (0.840-1.033)	0.177
South	1.068 (0.983-1.161)	0.120											1.092 (1.005-1.188)	0.039
West	1.220 (1.112-1.339)	<0.001											1.139 (1.036-1.252)	0.007
Metastatic disease	2.000 (1.868-2.142)	<0.001											1.999 (1.866-2.140)	<0.001
Non-metropolitan status (versus metropolitan)			0.742 (0.681-0.808)	<0.001										
County-level median household income (per $10,000)					1.067 (1.050-1.083)	<0.001							1.045 (1.021-1.070)	<0.001
County-level post-high school education							1.014 (1.010-1.017)	<0.001					1.005 (0.999-1.010)	0.054
County-level median distance to ED (per mile)									0.973 (0.962-0.984)	<0.001			0.982 (0.970-0.994)	0.003
County-level median home value (per $10,000)											1.007 (1.005-1.008)	<0.001		

aOR, Adjusted Odds Ratio; CI, Confidence Interval; ED, Emergency Department; CCI, Charlson Comorbidity Index.

## 4 Discussion

This study examined demographic, clinical, and county-level contextual factors associated with claims-identified clinical research participation among Medicare FFS beneficiaries aged 65 years and older with prostate cancer. Overall, documented clinical research participation was uncommon and was associated with several patient- and county-level characteristics. These findings suggest that participation in clinical research is influenced not only by patient-level characteristics but also by the broader community context in which beneficiaries reside.

Several patient-level characteristics were independently associated with documented clinical research participation. Consistent with prior literature, White beneficiaries were more likely to have evidence of clinical research participation than non-White beneficiaries, reflecting persistent racial disparities in prostate cancer research despite ongoing efforts to improve diversity (9, 10). Although some evidence suggests modest improvements in participation among Hispanic populations over time (9), racial and ethnic disparities in clinical research remain well documented and continue to raise concerns regarding the representativeness and generalizability of clinical research.

Clinical characteristics were also associated with documented clinical research participation. Beneficiaries with metastatic disease had higher odds of clinical research participation than beneficiaries without metastatic disease. This finding is consistent with prior studies reporting greater participation in clinical trials among patients with advanced disease compared to those with localized disease (26). This association may reflect the growing availability of clinical trials evaluating therapies for advanced-stage disease given increases in new therapies for metastatic disease (27, 28), coupled with more frequent engagement with oncology specialists among patients with advanced disease (29, 30). However, eligibility criteria for certain studies continue to exclude patients with specific comorbidities or disease characteristics (31), potentially limiting research opportunities for subsets of this population. This remains an important consideration because participation in therapeutic clinical trials has been associated with improved survival outcomes among patients with metastatic prostate cancer receiving first-line chemotherapy (32). However, because clinical research participation in the present study was identified using ICD-10-CM diagnosis code Z00.6, these findings should be interpreted as associations with documented clinical research participation rather than confirmed enrollment in prostate cancer-specific clinical trials.

In contrast, the positive association between higher CCI scores and documented clinical research participation differs from previous studies, which have generally reported lower enrollment among patients with greater comorbidity burden (33, 34). One plausible explanation is that this finding reflects characteristics of administrative claims data rather than preferential enrollment of medically complex patients. Beneficiaries with more frequent healthcare encounters have greater opportunities for documentation of comorbid conditions as well as increased interactions with treating clinicians, specialists, and healthcare systems where clinical research opportunities may be discussed and research-related services may be documented using ICD-10-CM diagnosis code Z00.6. Consistent with this interpretation, continuity of care and greater engagement with healthcare providers have previously been associated with increased clinical trial participation (35), suggesting that greater engagement with the healthcare system may increase awareness of, referral to, and participation in clinical research. Accordingly, the observed association between higher CCI scores and documented clinical research participation should not be interpreted as evidence that patients with greater comorbidity burden are inherently more likely to participate in clinical research. Looking ahead, efforts to broaden clinical trial eligibility criteria, including the U.S. Food and Drug Administration’s 2024 Action Plan (36) and initiatives to reduce exclusions based on age and comorbid conditions, may improve opportunities for clinical research participation among medically complex patients in future studies.

Beyond patient characteristics, this study also demonstrated that county-level socioeconomic context and healthcare accessibility were consistently associated with clinical research participation. Beneficiaries with documented clinical research participation were more likely to reside in metropolitan counties and in counties with shorter median distances to the nearest emergency and urgent care facilities. Because the AHRQ SDOH Database does not include county-level measures of proximity to clinical research sites, these distance measures were used as contextual indicators of general healthcare accessibility. Accordingly, the observed associations should be interpreted as reflecting differences in county-level healthcare infrastructure rather than a direct measure of access to clinical research. Nonetheless, these findings align with prior studies demonstrating geographic variation in the availability of cancer centers and clinical research infrastructure. For example, U.S. counties with a higher proportion of Black residents are less likely to have a cancer center than counties with a lower proportion of Black residents, and when such centers exist, they offer fewer cancer clinical trials per capita per year (37). Collectively, these findings suggest that county-level healthcare infrastructure may influence opportunities for clinical research participation and underscore the importance of addressing barriers to improve equitable participation.

County-level socioeconomic context also played an important role. Beneficiaries with documented clinical research participation were more likely to reside in counties characterized by greater socioeconomic resources, including higher household incomes, lower unemployment and poverty rates, higher educational attainment, and higher housing values. Because these measures reflect county-level context rather than individual socioeconomic circumstances, the findings should be interpreted as associations between community characteristics and clinical research participation rather than individual-level differences. Nonetheless, these findings are consistent with prior studies demonstrating geographic variation in the distribution of cancer centers and clinical research infrastructure (37) and that prostate cancer trial participants more often reside in areas with greater socioeconomic resources (8). Together, these findings suggest that the broader community-level socioeconomic environment may influence opportunities for clinical research participation, even after accounting for individual clinical characteristics.

These findings reinforce that disparities in clinical research participation extend beyond individual patient characteristics and are shaped by broader structural and community-level factors. Previous studies have identified barriers to clinical research participation, including logistical challenges, financial concerns, limited research infrastructure, and medical mistrust (38–40). Strategies such as expanding recruitment efforts, providing transportation, reducing logistical and financial burdens, and incorporating community perspectives into clinical trial design have demonstrated promise in improving participation among historically underrepresented populations (41–43). Additionally, efforts such as community outreach, recruitment through safety-net health systems, and the engagement of patient advocates and navigators have shown promise in increasing clinical trial participation (41, 44–47). Although the present study cannot evaluate the effectiveness of specific interventions, the observed associations between county-level socioeconomic context, healthcare accessibility, and documented clinical research participation support continued efforts to reduce structural barriers and promote equitable opportunities for participation in clinical research.

This study has several strengths. It leveraged a large national sample of Medicare FFS beneficiaries with prostate cancer, providing substantial statistical power to evaluate demographic, clinical, and contextual factors associated with claims-based clinical research participation among older adults. Additionally, linkage of Medicare claims with the AHRQ SDOH database enabled evaluation of county-level contextual characteristics across multiple SDOH domains, allowing assessment of both patient- and community-level factors associated with research participation. This multilevel framework provides a more comprehensive assessment of factors associated with clinical research participation than studies limited to individual-level demographic or clinical characteristics alone and is consistent with established health equity frameworks recognizing that participation in clinical research is influenced by both individual characteristics and the broader social, structural, and healthcare environments in which patients reside.

However, several limitations should also be considered when interpreting these findings. First, the study population was restricted to Medicare FFS beneficiaries aged 65 years and older, limiting the generalizability of the findings to younger patients, beneficiaries enrolled in Medicare Advantage, Medicaid, or commercial insurance, or individuals without insurance. Second, clinical research participation was identified using ICD-10-CM diagnosis code Z00.6, a claims-based diagnosis code that captures healthcare encounters associated with clinical research programs. Although this code is required by The Centers for Medicare & Medical Services (CMS) on research-related healthcare claims, it is not specific to prostate cancer and does not distinguish participation in prostate cancer-specific studies from participation in research involving other conditions. In addition, the code does not identify the type, phase, or duration of the research program. Consequently, some beneficiaries identified using Z00.6 may have participated in clinical research unrelated to their prostate cancer, and the outcome should therefore be interpreted as clinical research participation among Medicare beneficiaries with prostate cancer rather than confirmed participation in prostate cancer-specific clinical trials. Future studies linking administrative claims with clinical trial registries, cancer registries, or electronic health records would enable more precise characterization of research participation.

Additionally, administrative claims data may lack clinical granularity, and certain factors that may influence eligibility for clinical research participation, including disease severity or tumor stage, are not captured, limiting the ability to fully adjust for clinical characteristics associated with participation. Similarly, claims-based measures of comorbidity may be influenced by healthcare utilization, which may partially explain the observed association between higher CCI scores and documented clinical research participation.

In addition, county-level SDOH measures represent contextual characteristics rather than individual-level socioeconomic circumstances and therefore may not fully capture within-county heterogeneity in healthcare access, transportation, financial resources, or community support. Accordingly, the observed associations should be interpreted at the county level, and individual-level inferences should be avoided because of the potential for ecological fallacy. Furthermore, the relatively small number of non-White beneficiaries with documented clinical research participation limited the ability to conduct detailed subgroup analyses or evaluate temporal trends by race. Finally, the study period (2016-2021) included the COVID-19 pandemic, which substantially disrupted cancer care delivery, clinical research operations, and patient access to healthcare services nationwide. Because temporal analyses were beyond the scope of this study, the potential impact of these disruptions on patterns of clinical research participation could not be evaluated.

## 5 Conclusion

Overall, documented clinical research participation among Medicare FFS beneficiaries with prostate cancer was associated with several demographic, clinical, and county-level SDOH factors. These findings underscore ongoing disparities in access to clinical research and highlight the influence of community-level socioeconomic context and healthcare accessibility. Efforts to promote more equitable participation in clinical research may inform future strategies that address structural barriers, strengthen research infrastructure, and improve the systematic collection of demographic and SDOH data to monitor progress toward more representative research enrollment. Stakeholders may also consider investing in community partnerships, outreach, and research infrastructure to expand opportunities for participation among populations that have been historically underrepresented in clinical research. Future studies linking administrative claims with clinical trial registries or other clinical data sources are needed to better characterize prostate cancer-specific research participation, validate claims-based measures of clinical research participation, and evaluate the effectiveness of targeted interventions to improve equitable opportunities for participation in clinical research.

## Data Availability

This study used administrative claims data from The Centers for Medicare & Medicaid Services (CMS). Due to data use agreements signed with CMS, the data cannot be provided externally. Other researchers can purchase the same dataset to carry out similar analyses. Requests to access the datasets should be directed to https://www.cms.gov/.
