# Supplementary material for: Factors associated with clinical research participation among Medicare beneficiaries with prostate cancer in the United States
Source: Front Oncol. 2026 Sep 3;16:1887816. doi: 10.3389/fonc.2026.1887816 (PMC13583424; doi:10.3389/fonc.2026.1887816)
Supplement: Supplementary file 1 [file Table1.docx]

Supplemental Table 1. ICD-10 diagnosis codes for metastatic disease used together with ICD-10 diagnosis code C61 (Malignant neoplasm of prostate)

| ICD-10 Code | Description |
| --- | --- |
| C77.0 | Secondary and unspecified malignant neoplasm of lymph nodes of head, face and neck |
| C77.1 | Secondary and unspecified malignant neoplasm of intrathoracic lymph nodes |
| C77.2 | Secondary and unspecified malignant neoplasm of intra-abdominal lymph nodes |
| C77.3 | Secondary and unspecified malignant neoplasm of axilla and upper limb lymph nodes |
| C77.4 | Secondary and unspecified malignant neoplasm of inguinal and lower limb lymph nodes |
| C77.5 | Secondary and unspecified malignant neoplasm of intrapelvic lymph nodes |
| C77.8 | Secondary and unspecified malignant neoplasm of lymph nodes of multiple regions |
| C77.9 | Secondary and unspecified malignant neoplasm of lymph node, unspecified |
| C78.0 | Secondary malignant neoplasm of lung [Non-Specific Code] |
| C78.1 | Secondary malignant neoplasm of mediastinum |
| C78.2 | Secondary malignant neoplasm of pleura |
| C78.3 | Secondary malignant neoplasm of other and unspecified respiratory organs [Non-Specific Code] |
| C78.39 | Secondary malignant neoplasm of other respiratory organs |
| C78.7 | Secondary malignant neoplasm of liver and intrahepatic bile duct |
| C78.8 | Secondary malignant neoplasm of other and unspecified digestive organs [Non-Specific Code] |
| C79.00 | Secondary malignant neoplasm of unspecified kidney and renal pelvis |
| C79.11 | Secondary malignant neoplasm of bladder |
| C79.2 | Secondary malignant neoplasm of skin |
| C79.31 | Secondary malignant neoplasm of brain |
| C79.32 | Secondary malignant neoplasm of cerebral meninges |
| C79.40 | Secondary malignant neoplasm of unspecified part of nervous system |
| C79.49 | Secondary malignant neoplasm of other parts of nervous system |
| C79.51 | Secondary malignant neoplasm of bone |
| C79.52 | Secondary malignant neoplasm of bone marrow |
| C79.82 | Secondary malignant neoplasm of genital organs |
| C79.89 | Secondary malignant neoplasm of other specified sites |
| C79.9 | Secondary malignant neoplasm of unspecified site |
| C80.0 | Disseminated malignant neoplasm, unspecified |

**Abbreviations:** ICD-10 = International Classification of Diseases, 10th Revision

Legend: This list of codes comes from: Dolan MT, Kim S, Shao Y-H, Lu-Yao GL. Authentication of Algorithm to Detect Metastases in Men with Prostate Cancer Using ICD‐9 Codes. Epidemiology Res Int. 2012;2012:970406. https://doi.org/10.1155/2012/970406
